# (2,7-Dimethoxy­naphthalene-1,8-di­yl)bis­(4-fluoro­benzo­yl)dimethanone

**DOI:** 10.1107/S1600536810000486

**Published:** 2010-01-09

**Authors:** Shoji Watanabe, Atsushi Nagasawa, Akiko Okamoto, Keiichi Noguchi, Noriyuki Yonezawa

**Affiliations:** a Department of Organic and Polymer Materials Chemistry, Tokyo University of Agriculture & Technology, Koganei, Tokyo 184-8588, Japan; b Instrumentation Analysis Center, Tokyo University of Agriculture & Technology, Koganei, Tokyo 184-8588, Japan

## Abstract

The title compound, C_26_H_18_F_2_O_4_, is a naphthalene derivative in which the two aroyl groups at the 1- and 8-positions (*peri* positions) are *anti* to each other. There is an appreciable difference in the dihedral angles between the naphthalene ring system and the two benzene rings [66.88 (7)° and 88.09 (6)°]. In the crystal, weak C—H⋯O inter­actions involving one of the carbonyl groups and an aromatic C—H group *ortho* to the F atom seem to stabilize the packing of the mol­ecules.

## Related literature

Our study on the selective electrophilic aromatic aroylation of 2,7-dimethoxy­naphthalene, has shown *peri*-aroylnaphthalene compounds to be formed regioselectively with the aid of a suitable acidic mediator, see: (Okamoto & Yonezawa, 2009 ▶). For related structures, see: Nakaema *et al.* (2007 ▶, 2008 ▶); Mitsui *et al.* (2009 ▶).
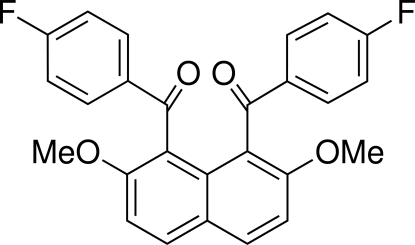


## Experimental

### 

#### Crystal data

C_26_H_18_F_2_O_4_
*M* *_r_* = 432.42
Monoclinic, 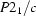

*a* = 9.87444 (18) Å
*b* = 17.0275 (3) Å
*c* = 14.9671 (3) Å
β = 126.871 (1)°
*V* = 2013.19 (7) Å^3^
*Z* = 4
Cu *K*α radiation
μ = 0.91 mm^−1^
*T* = 296 K
0.40 × 0.40 × 0.10 mm

#### Data collection

Rigaku R-AXIS RAPID diffractometer
Absorption correction: numerical (*NUMABS*; Higashi, 1999 ▶) *T*_min_ = 0.713, *T*_max_ = 0.915
36825 measured reflections
3682 independent reflections
3338 reflections with *I* > 2σ(*I*)
*R*_int_ = 0.030

#### Refinement

*R*[*F* ^2^ > 2σ(*F* ^2^)] = 0.034
*wR*(*F* ^2^) = 0.095
*S* = 1.07
3682 reflections
314 parameters
H atoms treated by a mixture of independent and constrained refinement
Δρ_max_ = 0.22 e Å^−3^
Δρ_min_ = −0.18 e Å^−3^

### 

Data collection: *PROCESS-AUTO* (Rigaku, 1998 ▶); cell refinement: *PROCESS-AUTO*; data reduction: *CrystalStructure* (Rigaku/MSC, 2004 ▶); program(s) used to solve structure: *SIR2004* (Burla *et al.*, 2005 ▶); program(s) used to refine structure: *SHELXL97* (Sheldrick, 2008 ▶); molecular graphics: *ORTEP* (Burnett & Johnson, 1996 ▶); software used to prepare material for publication: *SHELXL97*.

## Supplementary Material

Crystal structure: contains datablocks I, global. DOI: 10.1107/S1600536810000486/ds2015sup1.cif

Structure factors: contains datablocks I. DOI: 10.1107/S1600536810000486/ds2015Isup2.hkl

Additional supplementary materials:  crystallographic information; 3D view; checkCIF report

## Figures and Tables

**Table 1:** Hydrogen-bond geometry (Å, °)

*D*—H⋯*A*	*D*—H	H⋯*A*	*D*⋯*A*	*D*—H⋯*A*
C16—H7⋯O1^i^	1.00	2.54	3.493 (2)	158
C21—H10⋯O1^ii^	0.99	2.68	3.636 (2)	161

Symmetry codes: (i) 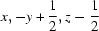; (ii) 
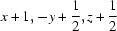
.

**Fig. 1.**
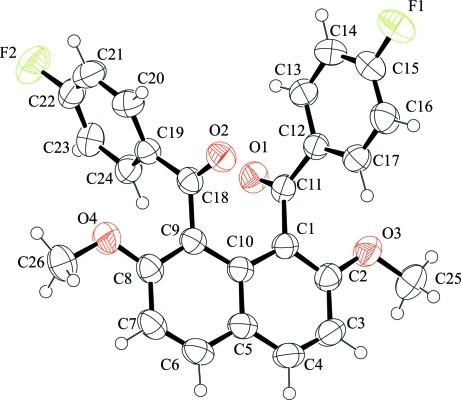
Molecular structure with displacement ellipsoids at 50% probability for non-H atoms.

**Fig. 2.**
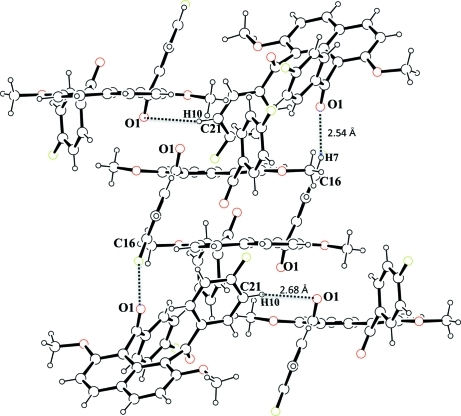
Weak C–H···O interactions (dotted lines).

## supplementary crystallographic information

### Comment

In the course of our study on selective electrophilic aromatic aroylation of 2,7-dimethoxynaphthalene, *peri*-aroylnaphthalene compounds have proved to be formed regioselectively with the aid of suitable acidic mediator (Okamoto & Yonezawa, 2009). The aroyl groups at 1,8-positions of the naphthalene rings in these compounds are oriented in opposite fashion and are found to be non-coplanar resulting in partial disruption in π-conjugation systems. Recently, we have reported the X-ray crystal structures of 1,8-bis(4-chlorobenzoyl)-2,7-dimethoxynaphthalene (Nakaema *et al.*,  2007) and 1,8-dibenzoyl-2,7-dimethoxynaphthalene (Nakaema *et al.*,  2008). As a part of the course of our continuous study on the molecular structures of this kind of homologous molecules, the X-ray crystal structure of title compound, *peri*-aroylnaphthalene bearing fluoro groups, is discussed in this report.

In the molecule (Fig. 1), the dihedral angle between benzene rings [C12–C17] and [C19–C24] is 32.34 (8)°, which is distinctively larger than that of 1,8-bis(4-chlorobenzoyl)-2,7-dimethoxynaphthalene [7.99 (8)°]. The dihedral angles between the naphthalene ring [C1–C10] plane and the planes of two benzene rings [C12–C17] and [C19–C24] are 66.88 (7)° and 88.12 (6)°, respectively. The difference between two dihedral angles is larger than that of the analogous compound, which has the corresponding angles of 71.98 (7)° and 71.55 (7)°.

The molecules are packed in the crystal lattice apparently stabilized by C—H···O interactions inovolving C16, C21 and O1 [2.54 Å, 158° and 2.68 Å, 161°](Fig. 2 and Table 1).

### Experimental

To a 10 ml flask, 4-fluorobenzoic acid (4.4 mmol, 616.5 mg) and phosphorus pentoxide–methanesulfonic acid (P_2_O_5_–MsOH; 8.8 ml) were placed and stirred at 60°C. To the reaction mixture thus obtained, 2,7-dimethoxynaphthalene (2.0 mmol, 376.4 mg) was added. After the reaction mixture was stirred at 60 °C for 1 h, it was poured into ice-cold water (10 ml) and the mixture was extracted with CHCl_3_ (10 ml × 3). The combined extracts were washed with 2 *M* aqueous NaOH followed by washing with brine. The organic layers thus obtained were dried over anhydrous MgSO_4_. The solvent was removed under reduced pressure to give cake (98% yield). Crude product was purified by recrystallization from EtOH (77% isolated yield). Furthermore, the isolated product was crystallized from toluene–hexane to give single-crystal.

Spectroscopic Data:

^1^H NMR δ (300 MHz, CDCl_3_): 3.70 (6*H*, s), 7.02 (4*H*, dd, *J* = 8.6 Hz), 7.21 (2*H*, d, *J *= 8.7 Hz), 7.71 (4*H*, dd, *J* = 8.41 Hz), 7.96 (2*H*, d, *J* = 8.7 Hz). ^13^C NMR δ (300 MHz, CDCl_3_): 56.289, 111.12, 114.92, 115.21, 125.47, 129.76, 131.52, 131.64, 132.25, 135.19, 156.24, 163.81, 167.17, 195.38. IR (KBr): 1596 (C=O), 1270 (Ar–O–Me). m.p. = 196°C. Anal. Calcd for C_26_H_18_F_2_O_4_; C, 70.27; H, 4.20. Found C, 72.05; H, 4.20.

### Refinement

All the H atoms were found in difference maps and were subsequently refined as riding atoms, with C—H = 0.93 (aromatic) and 0.96 (methyl) Å, and *U*_iso_(H) = 1.2*U*_eq_(C).

### Crystal data

**Table:**

C_26_H_18_F_2_O_4_	*F*(000) = 896
*M**_r_* = 432.42	*D*_x_ = 1.427 Mg m^−^^3^
Monoclinic, *P*2_1_/*c*	Melting point: 469 K
Hall symbol: -P 2ybc	Cu *K*α radiation, λ = 1.54187 Å
*a* = 9.87444 (18) Å	Cell parameters from 33844 reflections
*b* = 17.0275 (3) Å	θ = 3.7–68.2°
*c* = 14.9671 (3) Å	µ = 0.91 mm^−^^1^
β = 126.871 (1)°	*T* = 296 K
*V* = 2013.19 (7) Å^3^	Platelet, yellow
*Z* = 4	0.40 × 0.40 × 0.10 mm

### Data collection

**Table:**

Rigaku R-AXIS RAPID diffractometer	3682 independent reflections
Radiation source: fine-focus sealed tube	3338 reflections with *I* > 2σ(*I*)
graphite	*R*_int_ = 0.030
Detector resolution: 10.00 pixels mm^-1^	θ_max_ = 68.2°, θ_min_ = 4.5°
ω scans	*h* = −11→11
Absorption correction: numerical (*NUMABS*; Higashi, 1999)	*k* = −20→20
*T*_min_ = 0.713, *T*_max_ = 0.915	*l* = −18→18
36825 measured reflections	

### Refinement

**Table:**

Refinement on *F*^2^	Secondary atom site location: difference Fourier map
Least-squares matrix: full	Hydrogen site location: inferred from neighbouring sites
*R*[*F*^2^ > 2σ(*F*^2^)] = 0.034	H atoms treated by a mixture of independent and constrained refinement
*wR*(*F*^2^) = 0.095	*w* = 1/[σ^2^(*F*_o_^2^) + (0.0494*P*)^2^ + 0.3061*P*] where *P* = (*F*_o_^2^ + 2*F*_c_^2^)/3
*S* = 1.07	(Δ/σ)_max_ < 0.001
3682 reflections	Δρ_max_ = 0.22 e Å^−^^3^
314 parameters	Δρ_min_ = −0.18 e Å^−^^3^
0 restraints	Extinction correction: *SHELXL97* (Sheldrick, 2008), Fc^*^=kFc[1+0.001xFc^2^λ^3^/sin(2θ)]^-1/4^
Primary atom site location: structure-invariant direct methods	Extinction coefficient: 0.0030 (3)

### Special details

**Table:**

Geometry. All e.s.d.'s (except the e.s.d. in the dihedral angle between two l.s. planes) are estimated using the full covariance matrix. The cell e.s.d.'s are taken into account individually in the estimation of e.s.d.'s in distances, angles and torsion angles; correlations between e.s.d.'s in cell parameters are only used when they are defined by crystal symmetry. An approximate (isotropic) treatment of cell e.s.d.'s is used for estimating e.s.d.'s involving l.s. planes.
Refinement. Refinement of *F*^2^ against ALL reflections. The weighted *R*-factor *wR* and goodness of fit *S* are based on *F*^2^, conventional *R*-factors *R* are based on *F*, with *F* set to zero for negative *F*^2^. The threshold expression of *F*^2^ > σ(*F*^2^) is used only for calculating *R*-factors(gt) *etc*. and is not relevant to the choice of reflections for refinement. *R*-factors based on *F*^2^ are statistically about twice as large as those based on *F*, and *R*- factors based on ALL data will be even larger.

### Fractional atomic coordinates and isotropic or equivalent isotropic displacement parameters (Å^2^)

**Table:**

	*x*	*y*	*z*	*U*_iso_*/*U*_eq_	
F1	−0.21053 (13)	0.44424 (5)	−0.09780 (9)	0.0824 (3)	
F2	0.35965 (13)	0.21569 (6)	0.65179 (7)	0.0878 (3)	
O1	−0.24490 (12)	0.16209 (5)	0.15843 (7)	0.0570 (2)	
O2	0.09608 (12)	0.18156 (5)	0.15466 (8)	0.0598 (2)	
O3	−0.55074 (11)	0.11265 (6)	−0.11168 (8)	0.0634 (3)	
O4	0.31918 (12)	0.01602 (6)	0.31605 (8)	0.0684 (3)	
C1	−0.26555 (15)	0.08412 (7)	0.02104 (9)	0.0444 (3)	
C2	−0.42192 (16)	0.05978 (7)	−0.07141 (10)	0.0503 (3)	
C3	−0.44496 (18)	−0.01516 (8)	−0.11871 (11)	0.0587 (3)	
H1	−0.5531	−0.0296	−0.1825	0.070*	
C4	−0.31155 (19)	−0.06521 (7)	−0.07028 (11)	0.0580 (3)	
H2	−0.3237	−0.1170	−0.1017	0.070*	
C5	−0.15072 (17)	−0.04486 (7)	0.02702 (10)	0.0501 (3)	
C6	−0.01766 (19)	−0.10000 (7)	0.08034 (12)	0.0574 (3)	
H3	−0.0389	−0.1520	0.0463	0.067*	
C7	0.13559 (18)	−0.08282 (8)	0.17722 (11)	0.0573 (3)	
H4	0.2247	−0.1225	0.2143	0.071*	
C8	0.16525 (16)	−0.00701 (7)	0.22300 (10)	0.0516 (3)	
C9	0.04104 (15)	0.05019 (7)	0.17254 (9)	0.0450 (3)	
C10	−0.12396 (15)	0.03188 (7)	0.07386 (9)	0.0438 (3)	
C11	−0.25571 (14)	0.16202 (7)	0.07300 (9)	0.0444 (3)	
C12	−0.25352 (14)	0.23685 (7)	0.02252 (9)	0.0449 (3)	
C13	−0.20106 (18)	0.30478 (8)	0.08654 (11)	0.0559 (3)	
H5	−0.1715	0.3006	0.1573	0.068*	
C14	−0.1873 (2)	0.37483 (8)	0.04636 (12)	0.0631 (4)	
H6	−0.1474	0.4221	0.0902	0.077*	
C15	−0.22767 (18)	0.37560 (8)	−0.05889 (12)	0.0592 (3)	
C16	−0.28166 (19)	0.31051 (8)	−0.12562 (11)	0.0614 (3)	
H7	−0.3057	0.3146	−0.2008	0.086*	
C17	−0.29344 (17)	0.24052 (7)	−0.08343 (10)	0.0526 (3)	
H8	−0.3335	0.1938	−0.1306	0.059*	
C18	0.09933 (14)	0.13262 (7)	0.21535 (10)	0.0461 (3)	
C19	0.16719 (14)	0.15291 (7)	0.33199 (10)	0.0459 (3)	
C20	0.29027 (17)	0.21100 (8)	0.38710 (12)	0.0576 (3)	
H9	0.3323	0.2342	0.3471	0.074*	
C21	0.35501 (18)	0.23239 (9)	0.49493 (12)	0.0642 (4)	
H10	0.4460	0.2721	0.5367	0.088*	
C22	0.29323 (17)	0.19592 (8)	0.54466 (11)	0.0598 (4)	
C23	0.17105 (17)	0.13886 (9)	0.49316 (11)	0.0597 (3)	
H11	0.1302	0.1174	0.5288	0.071*	
C24	0.10868 (16)	0.11671 (8)	0.38576 (10)	0.0527 (3)	
H12	0.0217	0.0758	0.3467	0.062*	
C25	−0.71784 (19)	0.08920 (13)	−0.20143 (14)	0.0706 (4)	
C26	0.4565 (2)	−0.03775 (11)	0.36866 (15)	0.0697 (4)	
H13	−0.751 (3)	0.0383 (12)	−0.1820 (16)	0.098 (6)*	
H14	−0.786 (3)	0.1326 (12)	−0.2121 (15)	0.088 (6)*	
H15	−0.728 (2)	0.0805 (11)	−0.2710 (16)	0.094 (6)*	
H16	0.429 (2)	−0.0860 (11)	0.3956 (14)	0.081 (5)*	
H17	0.556 (3)	−0.0075 (11)	0.4306 (16)	0.094 (6)*	
H18	0.479 (2)	−0.0552 (10)	0.3166 (15)	0.082 (5)*	

### Atomic displacement parameters (Å^2^)

**Table:**

	*U*^11^	*U*^22^	*U*^33^	*U*^12^	*U*^13^	*U*^23^
F1	0.1025 (7)	0.0541 (5)	0.1006 (7)	0.0052 (4)	0.0663 (6)	0.0177 (4)
F2	0.0833 (6)	0.1105 (8)	0.0550 (5)	−0.0057 (5)	0.0337 (5)	−0.0239 (5)
O1	0.0680 (6)	0.0644 (6)	0.0454 (5)	0.0048 (4)	0.0377 (4)	0.0000 (4)
O2	0.0666 (6)	0.0548 (5)	0.0592 (5)	−0.0018 (4)	0.0384 (5)	0.0083 (4)
O3	0.0468 (5)	0.0667 (6)	0.0569 (5)	−0.0010 (4)	0.0206 (4)	−0.0085 (4)
O4	0.0548 (5)	0.0657 (6)	0.0631 (6)	0.0165 (4)	0.0237 (5)	0.0001 (5)
C1	0.0507 (6)	0.0438 (6)	0.0414 (6)	−0.0025 (5)	0.0291 (5)	−0.0006 (5)
C2	0.0523 (7)	0.0531 (7)	0.0445 (6)	−0.0034 (5)	0.0285 (6)	−0.0011 (5)
C3	0.0631 (8)	0.0568 (7)	0.0490 (7)	−0.0149 (6)	0.0297 (6)	−0.0093 (6)
C4	0.0771 (9)	0.0442 (7)	0.0547 (7)	−0.0094 (6)	0.0406 (7)	−0.0078 (5)
C5	0.0678 (8)	0.0425 (6)	0.0498 (6)	−0.0030 (5)	0.0405 (6)	−0.0011 (5)
C6	0.0821 (9)	0.0425 (6)	0.0613 (8)	0.0044 (6)	0.0503 (8)	0.0006 (6)
C7	0.0729 (9)	0.0493 (7)	0.0589 (8)	0.0160 (6)	0.0444 (7)	0.0089 (6)
C8	0.0579 (7)	0.0525 (7)	0.0489 (6)	0.0078 (6)	0.0345 (6)	0.0051 (5)
C9	0.0527 (7)	0.0435 (6)	0.0443 (6)	0.0026 (5)	0.0321 (5)	0.0020 (5)
C10	0.0548 (7)	0.0418 (6)	0.0416 (6)	−0.0004 (5)	0.0325 (5)	0.0016 (5)
C11	0.0403 (6)	0.0519 (7)	0.0381 (6)	0.0034 (5)	0.0218 (5)	−0.0015 (5)
C12	0.0437 (6)	0.0462 (6)	0.0427 (6)	0.0053 (5)	0.0248 (5)	−0.0016 (5)
C13	0.0647 (8)	0.0530 (7)	0.0477 (7)	0.0040 (6)	0.0325 (6)	−0.0046 (5)
C14	0.0718 (9)	0.0458 (7)	0.0674 (8)	0.0003 (6)	0.0395 (7)	−0.0074 (6)
C15	0.0637 (8)	0.0480 (7)	0.0692 (8)	0.0074 (6)	0.0417 (7)	0.0103 (6)
C16	0.0732 (9)	0.0610 (8)	0.0541 (7)	0.0048 (7)	0.0405 (7)	0.0063 (6)
C17	0.0621 (8)	0.0508 (7)	0.0463 (6)	0.0010 (6)	0.0332 (6)	−0.0035 (5)
C18	0.0426 (6)	0.0462 (6)	0.0491 (6)	0.0034 (5)	0.0272 (5)	0.0038 (5)
C19	0.0427 (6)	0.0425 (6)	0.0486 (6)	0.0023 (5)	0.0253 (5)	0.0003 (5)
C20	0.0559 (7)	0.0527 (7)	0.0665 (8)	−0.0071 (6)	0.0380 (7)	−0.0078 (6)
C21	0.0566 (8)	0.0597 (8)	0.0686 (9)	−0.0104 (6)	0.0334 (7)	−0.0187 (7)
C22	0.0516 (7)	0.0667 (8)	0.0469 (7)	0.0060 (6)	0.0220 (6)	−0.0099 (6)
C23	0.0537 (7)	0.0735 (9)	0.0485 (7)	0.0017 (6)	0.0288 (6)	0.0034 (6)
C24	0.0478 (6)	0.0552 (7)	0.0477 (6)	−0.0050 (5)	0.0246 (5)	0.0001 (5)
C25	0.0494 (8)	0.0904 (12)	0.0578 (9)	−0.0087 (8)	0.0247 (7)	−0.0112 (8)
C26	0.0586 (9)	0.0752 (10)	0.0733 (10)	0.0202 (8)	0.0385 (8)	0.0156 (8)

### Geometric parameters (Å, °)

**Table:**

F1—C15	1.3610 (15)	C12—C13	1.3888 (17)
F2—C22	1.3611 (15)	C13—C14	1.379 (2)
O1—C11	1.2176 (14)	C13—H5	0.9168
O2—C18	1.2188 (14)	C14—C15	1.374 (2)
O3—C2	1.3681 (16)	C14—H6	0.9600
O3—C25	1.4236 (17)	C15—C16	1.368 (2)
O4—C8	1.3651 (16)	C16—C17	1.3859 (19)
O4—C26	1.4197 (17)	C16—H7	1.0036
C1—C2	1.3808 (17)	C17—H8	0.9758
C1—C10	1.4299 (16)	C18—C19	1.4874 (16)
C1—C11	1.5110 (16)	C19—C24	1.3854 (17)
C2—C3	1.4100 (18)	C19—C20	1.3902 (17)
C3—C4	1.357 (2)	C20—C21	1.381 (2)
C3—H1	0.9430	C20—H9	0.9912
C4—C5	1.4099 (19)	C21—C22	1.363 (2)
C4—H2	0.9717	C21—H10	0.9902
C5—C6	1.4094 (18)	C22—C23	1.370 (2)
C5—C10	1.4305 (16)	C23—C24	1.3847 (18)
C6—C7	1.358 (2)	C23—H11	0.9157
C6—H3	0.9791	C24—H12	0.9809
C7—C8	1.4074 (18)	C25—H13	1.03 (2)
C7—H4	0.9755	C25—H14	0.95 (2)
C8—C9	1.3826 (17)	C25—H15	1.00 (2)
C9—C10	1.4302 (17)	C26—H16	1.018 (19)
C9—C18	1.5065 (16)	C26—H17	1.00 (2)
C11—C12	1.4881 (16)	C26—H18	0.975 (19)
C12—C17	1.3869 (16)		
			
C2—O3—C25	118.43 (12)	C13—C14—H6	122.3
C8—O4—C26	119.26 (12)	F1—C15—C16	118.51 (13)
C2—C1—C10	120.12 (11)	F1—C15—C14	118.29 (13)
C2—C1—C11	117.85 (11)	C16—C15—C14	123.19 (12)
C10—C1—C11	121.47 (10)	C15—C16—C17	117.81 (12)
O3—C2—C1	115.41 (11)	C15—C16—H7	119.3
O3—C2—C3	123.16 (11)	C17—C16—H7	122.8
C1—C2—C3	121.42 (12)	C16—C17—C12	121.05 (12)
C4—C3—C2	119.20 (12)	C16—C17—H8	118.7
C4—C3—H1	121.6	C12—C17—H8	120.3
C2—C3—H1	119.2	O2—C18—C19	120.52 (11)
C3—C4—C5	121.88 (12)	O2—C18—C9	119.18 (11)
C3—C4—H2	120.8	C19—C18—C9	120.24 (10)
C5—C4—H2	117.3	C24—C19—C20	119.52 (11)
C6—C5—C4	120.71 (11)	C24—C19—C18	121.83 (11)
C6—C5—C10	119.72 (12)	C20—C19—C18	118.64 (11)
C4—C5—C10	119.55 (12)	C21—C20—C19	120.52 (13)
C7—C6—C5	121.59 (12)	C21—C20—H9	122.1
C7—C6—H3	120.2	C19—C20—H9	117.3
C5—C6—H3	118.2	C22—C21—C20	118.23 (13)
C6—C7—C8	119.37 (12)	C22—C21—H10	120.0
C6—C7—H4	120.4	C20—C21—H10	121.7
C8—C7—H4	120.3	F2—C22—C21	118.26 (13)
O4—C8—C9	115.77 (11)	F2—C22—C23	118.51 (14)
O4—C8—C7	122.63 (11)	C21—C22—C23	123.20 (12)
C9—C8—C7	121.52 (12)	C22—C23—C24	118.31 (13)
C8—C9—C10	119.88 (11)	C22—C23—H11	120.2
C8—C9—C18	115.89 (11)	C24—C23—H11	121.5
C10—C9—C18	123.37 (10)	C23—C24—C19	120.21 (12)
C1—C10—C9	124.44 (10)	C23—C24—H12	120.4
C1—C10—C5	117.74 (11)	C19—C24—H12	119.4
C9—C10—C5	117.78 (11)	O3—C25—H13	110.7 (11)
O1—C11—C12	120.91 (10)	O3—C25—H14	104.1 (11)
O1—C11—C1	118.64 (11)	H13—C25—H14	113.3 (16)
C12—C11—C1	120.42 (9)	O3—C25—H15	111.1 (11)
C17—C12—C13	118.93 (11)	H13—C25—H15	108.7 (15)
C17—C12—C11	122.58 (10)	H14—C25—H15	109.0 (15)
C13—C12—C11	118.41 (10)	O4—C26—H16	110.5 (10)
C14—C13—C12	120.87 (12)	O4—C26—H17	105.2 (11)
C14—C13—H5	121.7	H16—C26—H17	113.2 (15)
C12—C13—H5	117.4	O4—C26—H18	110.6 (10)
C15—C14—C13	118.15 (12)	H16—C26—H18	108.1 (14)
C15—C14—H6	119.6	H17—C26—H18	109.3 (16)
			
C25—O3—C2—C1	−175.20 (12)	C10—C1—C11—O1	−68.78 (15)
C25—O3—C2—C3	3.86 (19)	C2—C1—C11—C12	−79.42 (14)
C10—C1—C2—O3	176.46 (10)	C10—C1—C11—C12	109.20 (12)
C11—C1—C2—O3	4.95 (16)	O1—C11—C12—C17	−168.99 (12)
C10—C1—C2—C3	−2.62 (18)	C1—C11—C12—C17	13.08 (17)
C11—C1—C2—C3	−174.13 (11)	O1—C11—C12—C13	14.35 (17)
O3—C2—C3—C4	−177.06 (12)	C1—C11—C12—C13	−163.58 (11)
C1—C2—C3—C4	2.0 (2)	C17—C12—C13—C14	−0.5 (2)
C2—C3—C4—C5	0.9 (2)	C11—C12—C13—C14	176.26 (12)
C3—C4—C5—C6	175.24 (12)	C12—C13—C14—C15	0.3 (2)
C3—C4—C5—C10	−2.9 (2)	C13—C14—C15—F1	−178.54 (13)
C4—C5—C6—C7	−176.63 (13)	C13—C14—C15—C16	0.4 (2)
C10—C5—C6—C7	1.52 (19)	F1—C15—C16—C17	178.04 (13)
C5—C6—C7—C8	−3.0 (2)	C14—C15—C16—C17	−0.9 (2)
C26—O4—C8—C9	−176.00 (12)	C15—C16—C17—C12	0.7 (2)
C26—O4—C8—C7	0.9 (2)	C13—C12—C17—C16	0.01 (19)
C6—C7—C8—O4	−175.71 (12)	C11—C12—C17—C16	−176.63 (12)
C6—C7—C8—C9	1.0 (2)	C8—C9—C18—O2	111.97 (13)
O4—C8—C9—C10	179.36 (10)	C10—C9—C18—O2	−57.38 (16)
C7—C8—C9—C10	2.44 (18)	C8—C9—C18—C19	−65.30 (14)
O4—C8—C9—C18	9.61 (16)	C10—C9—C18—C19	125.35 (12)
C7—C8—C9—C18	−167.31 (11)	O2—C18—C19—C24	150.27 (12)
C2—C1—C10—C9	−177.21 (11)	C9—C18—C19—C24	−32.49 (17)
C11—C1—C10—C9	−6.01 (17)	O2—C18—C19—C20	−28.41 (17)
C2—C1—C10—C5	0.53 (16)	C9—C18—C19—C20	148.83 (12)
C11—C1—C10—C5	171.73 (10)	C24—C19—C20—C21	0.4 (2)
C8—C9—C10—C1	173.95 (11)	C18—C19—C20—C21	179.11 (12)
C18—C9—C10—C1	−17.11 (17)	C19—C20—C21—C22	−0.9 (2)
C8—C9—C10—C5	−3.79 (16)	C20—C21—C22—F2	178.54 (12)
C18—C9—C10—C5	165.16 (11)	C20—C21—C22—C23	0.4 (2)
C6—C5—C10—C1	−176.01 (10)	F2—C22—C23—C24	−177.46 (12)
C4—C5—C10—C1	2.16 (16)	C21—C22—C23—C24	0.7 (2)
C6—C5—C10—C9	1.88 (16)	C22—C23—C24—C19	−1.2 (2)
C4—C5—C10—C9	−179.95 (11)	C20—C19—C24—C23	0.70 (19)
C2—C1—C11—O1	102.61 (13)	C18—C19—C24—C23	−177.97 (12)

### Hydrogen-bond geometry (Å, °)

**Table:**

*D*—H···*A*	*D*—H	H···*A*	*D*···*A*	*D*—H···*A*
C16—H7···O1^i^	1.00	2.54	3.493 (2)	158
C21—H10···O1^ii^	0.99	2.68	3.636 (2)	161

Symmetry codes: (i) *x*, −*y*+1/2, *z*−1/2; (ii) *x*+1, −*y*+1/2, *z*+1/2.
